# Prevalence and Risk Factors of Sarcopenia Among Community-Dwelling Older Adults in Nakhon Si Thammarat, Thailand

**DOI:** 10.3390/ijerph23081040

**Published:** 2026-08-10

**Authors:** Ratchaya Supalak, Naparat Doungchan, Arisa Sespheng, Kornanong Yuenyongchaiwat, Stephen J. Scholand, Chuchard Punsawad

**Affiliations:** 1 School of Medicine, Walailak University, Nakhon Si Thammarat 80160, Thailand; ratchaya.su@wu.ac.th (R.S.); naparat.do@wu.ac.th (N.D.); arisa.se@wu.ac.th (A.S.); 2 Center of Excellence in Tropical Pathobiology, Walailak University, Nakhon Si Thammarat 80160, Thailand; 3 Physiotherapy Department, Faculty of Allied Health Sciences, Thammasat University, Pathumthani 12120, Thailand; ykornano@tu.ac.th; 4 Thammasat University Research Unit for Physical Therapy, Thammasat University, Pathumthani 12120, Thailand; 5 Department of Medicine, University of Arizona, Tucson, AZ 85724, USA; rabiesfreeworld@yahoo.com

**Keywords:** sarcopenia, prevalence, risk factors, community-dwelling older adults

## Abstract

**Highlights:**

**Public health relevance—How does this work relate to a public health issue?**

- Sarcopenia is a growing public health concern in aging societies because it is associated with disability, falls, and reduced quality of life among older adults.
- This study highlights a high prevalence of sarcopenia (27.64%) among community-dwelling older adults in southern Thailand and identifies modifiable risk factors including physical activity, nutritional status, and BMI.

**Public health significance—Why is this work of significance to public health?**

- This study provides epidemiological evidence of sarcopenia among older Thai adults using the AWGS-2019 criteria, contributing to the regional data needed for aging-related health policies.
- Identifying socioeconomic and lifestyle-related determinants helps inform targeted prevention strategies to maintain functional independence and reduce the healthcare burden.

**Public health implications—What are the key implications or messages for practitioners, policy makers and/or researchers in public health?**

- Public health programs should promote regular physical activity and adequate protein-rich nutrition as key strategies to prevent sarcopenia in older adults.
- Community-based screening and early interventions should be integrated into primary healthcare systems to detect sarcopenia and malnutrition in aging populations.

**Abstract:**

Background: There is limited evidence regarding sarcopenia in Thailand, especially in southern provinces when applying the AWGS-2019 criteria. Objective: The present study aimed to determine the prevalence and independent risk factors of sarcopenia among community-dwelling older adults in Nakhon Si Thammarat. Methods: A cross-sectional study was conducted involving 246 adults aged 60 years or older. Sarcopenia was defined using the AWGS-2019 criteria. Data collection comprised the Mini Nutritional Assessment-Short Form (MNA-SF), Abbreviated Mental Test (AMT), and physical performance assessments. Multivariable logistic regression analysis was performed to identify independent predictors, incorporating both categorical and continuous variables. Results: The prevalence of sarcopenia was 27.64%. Multivariable analysis identified age of ≥80 years or older (AOR = 4.25, 95% CI: 1.12–16.14; *p* = 0.033) and body mass index (BMI) below 18.5 kg/m^2^ (AOR = 3.58, 95% CI: 1.20–10.65; *p* = 0.022) as significant risk factors. Protective factors included attainment of secondary education or higher (AOR = 0.12, 95% CI: 0.02–0.78; *p* = 0.025), BMI of 23.0 kg/m^2^ or greater (AOR = 0.19, 95% CI: 0.09–0.40; *p* < 0.001), engagement in regular physical activity (AOR = 0.21, 95% CI: 0.08–0.54; *p* = 0.001), and normal nutritional status (AOR = 0.24, 95% CI: 0.08–0.71; *p* = 0.010). Further continuous analysis confirmed that each 5-year increase in age significantly increased the risk of sarcopenia (AOR = 1.52, 95% CI: 1.14–2.03; *p* = 0.005), while each 1 kg/m^2^ increase in BMI was associated with a protective effect (AOR = 0.78, 95% CI: 0.69–0.88; *p* < 0.001). Conclusions: Sarcopenia is common among older adults in Southern Thailand. Both categorical and continuous analyses identify advanced age as a primary risk factor. In contrast, higher education, optimal body mass index, regular physical activity, and adequate nutrition serve as important protective factors. These results highlight the importance of community-based screening and targeted interventions that address nutritional adequacy and structured exercise to enhance quality of life in this population.

## 1. Introduction

Sarcopenia is a debilitating geriatric syndrome defined by the progressive loss of skeletal muscle mass, strength, and physical performance [1,2,3]. This condition is recognized as a critical public health concern among older adults because of its profound impact on functional independence and quality of life. Individuals with sarcopenia are at increased risk for adverse clinical outcomes, such as falls, fractures, disability, hospitalization, and mortality [4,5,6,7,8]. In addition to its clinical implications, sarcopenia imposes a substantial burden on healthcare systems, affecting patients, families, and the broader socioeconomic context. Current evidence indicates that sarcopenia is linked to significant healthcare expenditures worldwide [9,10,11,12,13].

The global prevalence of sarcopenia demonstrates considerable variation across ethnic groups, research environments, and diagnostic standards [13,14,15]. Among older adults worldwide, prevalence estimates range from 10–16% [13], but may increase to 20% to 30% in specific populations [14,15,16,17]. Thailand is experiencing a rapid demographic transition. In 2022, individuals aged 60 years and older comprised 20% of the population, marking the country’s official entry into a ‘complete aged society’ [18]. This demographic trend is expected to accelerate, with projections indicating that Thailand will attain ‘super-aged society’ status by 2031, when older adults are forecasted to represent 28% of the population [18,19]. These changes pose significant public health challenges, particularly the increasing burden of sarcopenia. Recent systematic reviews and meta-analyses have reported a pooled prevalence of sarcopenia among Thai older adults of 20.7% (95% CI: 14.4–27.8%), with a prevalence of 17.3% among community-dwelling individuals [16]. In the Thai population, key risk factors include advanced age, male sex, low body weight, and inadequate nutritional status [20,21,22,23].

Although several studies have investigated sarcopenia in Thailand [20,21,22,23], data specific to individual regions remain limited. This gap is especially evident in Nakhon Si Thammarat, a province with a rapidly aging population and unique socioeconomic and cultural factors influencing dietary habits and physical activity levels [24]. The lack of context-specific data impedes accurate assessment of the sarcopenia burden in Southern Thailand and limits the effectiveness of regional public health strategies. Furthermore, there is a notable deficiency in local research utilizing the updated 2019 Asian Working Group for Sarcopenia (AWGS-2019) criteria, which offer revised cutoff values appropriate for Asian populations [2]. Employing these updated criteria is essential for generating precise and clinically meaningful estimates to inform targeted sarcopenia prevention and rehabilitation initiatives in this region.

Given the substantial clinical overlap between sarcopenia and frailty, this study evaluated frailty status as an independent covariate within our multivariable modeling framework. While sarcopenia and frailty share an overlapping pathophysiological spectrum, they remain conceptually distinct but often coexist as heavily overlapping, co-occurring clinical states that operate within a syndemic cycle, sharing risk factors such as diminished physiological reserve [25,26,27]. In this study, frailty is conceptualized as a co-occurring clinical state of multi-system vulnerability. In rural settings, this state can act as a downstream amplifier of musculoskeletal decline, where systemic energy depletion and chronic subclinical inflammation accelerate muscle catabolism. Evaluating frailty in conjunction with sarcopenia enables a more comprehensive assessment of functional health and determines whether lifestyle and nutritional risk factors remain independent of overall frailty—even though the final adjusted association for frailty neared but did not cross the strict threshold for statistical significance (*p* = 0.068). Additionally, incorporating frailty status increases the clinical applicability of the findings, supporting improved risk stratification and geriatric care planning in community-dwelling populations [27].

Although research on sarcopenia is expanding, regional data in Thailand remain scarce, particularly in populous provinces such as Nakhon Si Thammarat. This province features a rapidly aging population with diverse cultural and lifestyle backgrounds that may distinctly influence muscle health. The absence of comprehensive local data conforming to the AWGS-2019 criteria constrains the formulation of effective public health strategies for healthy aging, sarcopenia prevention, and rehabilitation. Therefore, the present study assessed the prevalence and risk factors of sarcopenia among community-dwelling older adults in Nakhon Si Thammarat Province, Thailand, using the updated AWGS-2019 diagnostic criteria. In addition, we investigated the association between sarcopenia and frailty to more precisely characterize the functional health status of this population. We hypothesized that advanced chronological age, low Body Mass Index (BMI), low physical activity frequency, and poor nutritional status (malnutrition or risk of malnutrition) would be independently associated with a higher likelihood of sarcopenia, and that phenotypic frailty would be significantly more prevalent among individuals with sarcopenia compared to their robust counterparts. Specifically, we aimed to: (1) estimate the prevalence of sarcopenia among community-dwelling older adults in Southern Thailand using the updated AWGS-2019 diagnostic criteria; (2) identify independent sociodemographic, anthropometric, clinical, and lifestyle risk factors associated with this condition; and (3) examine the independent association between sarcopenia and frailty status within this rural population.

## 2. Materials and Methods

### 2.1. Study Design, Population and Sample Size Calculation

This cross-sectional study was conducted in Nakhon Si Thammarat Province, Southern Thailand. A multi-site purposive sampling strategy was employed to select three specific districts—Tha Sala, Pak Phanang, and Ron Phibun—to reflect distinct ecological, socioeconomic, and occupational characteristics. These districts were strategically chosen based on two primary criteria: (1): they represent a clear environmental gradient (coastal/semi-urban, lowland estuary/traditional, and inland/mountainous agricultural settings), and (2) they exhibit high demographic aging trends that meet or exceed regional averages, providing a robust framework to evaluate variations in muscle health [24].

Tha Sala District (N≈115,000; 16.8% aged ≥60 years) represents a coastal, semi-urbanized area undergoing rapid modernization and functioning as a regional educational hub. Pak Phanang District (N≈94,000; 21.2% aged ≥60 years) represents a traditional lowland estuary zone characterized by fishing and swiftlet farming, holding one of the highest aging indices in the province. Ron Phibun District (N≈83,000; 19.7% aged ≥ 60 years) represents an inland, mountainous agricultural zone where intensive physical labor in rubber and fruit plantations predominates. To ensure comparable healthcare access across these diverse environments and minimize systemic confounding, each district is similarly served by a secondary-level community hospital and a network of 12 to 15 primary care units.

The minimum required sample size was calculated using Lemeshow’s single-population proportion formula:

$$n=\frac{Z^{2}\cdot P\left(1-P\right)}{d^{2}}$$

where *n* is the minimum required sample size, *Z* is the standard normal deviation for a 95% confidence level (*Z* = 1.96), *P* is the estimated baseline prevalence of sarcopenia among Thai older adults, set at 20% (0.20) based on a recent systematic review [16], and *d* is the acceptable margin of error, set at 5% (0.05). Substitution of these parameters into the formula yielded a minimum required sample size of 246 participants:

$$n=\frac{{\left(1.96\right)}^{2}\cdot 0.20\cdot \left(1-0.20\right)}{{\left(0.05\right)}^{2}}=245.86\approx 246$$

Ultimately, a final sample of 246 eligible individuals was successfully recruited, providing sufficient statistical power for the analysis. Sarcopenia was screened and diagnosed in strict accordance with the 2019 Asian Working Group for Sarcopenia (AWGS-2019) consensus criteria [2].

### 2.2. Study Setting and Participant Recruitment

Data collection occurred between October and November 2025 in Nakhon Si Thammarat Province, Southern Thailand, a region undergoing a rapid demographic transition [18,24]. To maximize socio-ecological and occupational variability across the province—rather than relying on operational convenience—a deliberate multi-site purposive sampling strategy was employed. Three districts were strategically selected to reflect this diversity, capturing distinct coastal, river-basin, and inland agricultural environments: Tha Sala District, an urban-transition coastal area where modernization is reshaping health behaviors; Pak Phanang District, a traditional river-basin zone with distinct social and environmental vulnerabilities; and Ron Phibun District, an inland agricultural hub characterized by intensive physical labor and irregular sleep–wake patterns. This selection facilitated a nuanced assessment of how diverse environmental contexts affect muscle health, thereby capturing a diverse cross-section of lifestyle patterns to enhance the external validity of our model relative to a single-setting study.

**Figure 1:**
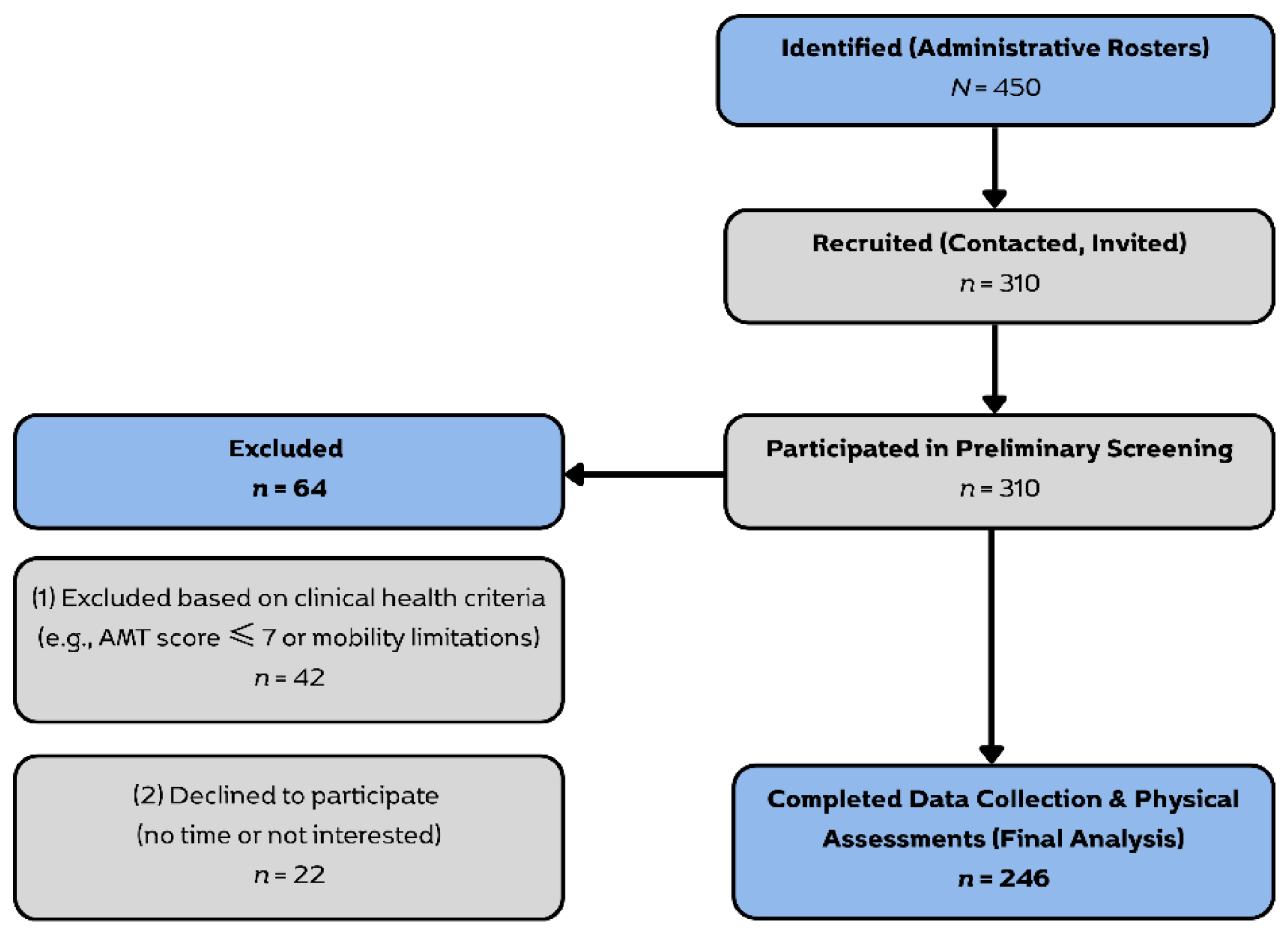
Participant flow diagram illustrating the recruitment and enrollment process based on STROBE guidelines. A total of 310 individuals were approached and screened from the administrative rosters. Following the screening process, 64 individuals were excluded, including 42 individuals who were excluded based on pre-defined clinical health criteria and 22 individuals who declined to participate. The final analysis included 246 participants, resulting in an overall participation rate of 79.4%.

The sampling frame consisted of older adults registered in the administrative rosters of five community health centers and three senior citizen clubs across the three selected districts, totaling approximately 450 individuals. A convenience sampling approach was utilized during monthly health promotion meetings, where all individuals present on the roster who met the initial age requirement (aged ≥ 60 years) were formally approached and invited to participate. To be eligible for inclusion, participants had to be aged 60 years or older and have maintained continuous residency in their respective districts for at least one year. To minimize participation barriers for less mobile individuals and mitigate potential selection bias, we collaborated closely with local Village Health Volunteers (VHVs) to facilitate and support transportation for elderly individuals with mobility limitations who wished to attend. Furthermore, all clinical assessment stations were localized inside neighborhood community health centers to reduce long-distance travel. Of the 310 individuals approached, 246 provided written informed consent and completed all assessments, resulting in a participation rate of 79.4% (Figure 1).

Among the 64 individuals who did not enroll, 42 were excluded based on pre-defined clinical health criteria. Specifically, cognitive function was screened using the Abbreviated Mental Test (AMT), which has been extensively validated for cognitive screening in older Thai populations (originally adapted by the Institute of Geriatric Medicine, Ministry of Public Health, Thailand). In accordance with Thai validation data, a cut-off score of ≤7 out of 10 points was strictly applied to exclude individuals with moderate-to-severe cognitive impairment who were unable to provide reliable self-reported lifestyle data or safely follow instructions for physical performance tests. Other explicit exclusion criteria included: (1) severe physical inability to perform the functional tests (e.g., being completely bedridden, having advanced osteoarthritis, or lower-limb amputations), and (2) a diagnosed terminal illness under palliative care (e.g., advanced-stage cancer). The remaining 22 individuals declined participation primarily due to time constraints or lack of interest. The final sample of 246 participants met the minimum size requirements calculated using Lemeshow’s formula, ensuring adequate statistical power. Although this multi-site approach captured diverse ecological contexts, the use of non-probability sampling and the high proportion of female participants may limit the generalizability of the findings to the broader male population or other regions of Southern Thailand. This gender imbalance primarily reflects the higher attendance of older women in community-based health activities in rural Thailand, which should be considered when interpreting the overall sarcopenia prevalence.

### 2.3. Data Collection and Measurement Instruments

Participant safety was strictly ensured prior to any physical testing by administering the Physical Activity Readiness Questionnaire for Everyone (2019 PAR-Q+) [28]. The PAR-Q+ was translated into Thai using a standard forward-back translation procedure and culturally adapted for Thai older adults. Content validity was evaluated by three health experts (IOC > 0.80), and internal consistency reliability yielded a Kuder–Richardson 20 (KR-20) coefficient of 0.76, indicating acceptable reliability in our sample. Only individuals cleared by this screening tool advanced to the physical performance assessments.

#### 2.3.1. General Information

Comprehensive sociodemographic data were collected through face-to-face structured interviews. The variables included sex, age, date of birth, educational attainment, marital status, monthly income, and occupation. These variables were chosen to generate a detailed baseline profile of the study population, which is essential for identifying potential confounding factors and elucidating the socioeconomic determinants of sarcopenia within the regional context.

#### 2.3.2. Health and Health Behaviors

The health status and lifestyle behaviors of participants were assessed using a 15-item instrument adapted from internationally recognized public health surveillance frameworks, specifically the World Health Organization (WHO) STEPwise Approach to NCD Surveillance (WHO STEPS) [29] and the U.S. National Health and Nutrition Examination Survey (NHANES) [30]. The instrument was translated and culturally adapted for the Thai context. Content validity was confirmed by three experts (IOC > 0.80), and the internal consistency in the current study demonstrated acceptable reliability with a Cronbach’s alpha of 0.79.

#### 2.3.3. Nutritional Status: Mini Nutritional Assessment–Short Form (MNA-SF)

Nutritional risk was evaluated using the Mini Nutritional Assessment–Short Form (MNA-SF). Previous research has reported internal consistency values (Cronbach’s alpha) ranging from 0.73 to 0.83 [31]. The MNA-SF has been widely validated for Thai older adults, and in the present study, the instrument demonstrated good internal reliability with a Cronbach’s alpha of 0.79. These psychometric properties support its use in both clinical diagnostics and community-based epidemiological studies.

#### 2.3.4. Frailty Screening

Frailty status was assessed using a modified version of Fried’s Frailty Phenotype model [25,32], which was operationally adapted for this community screening. This modified framework evaluated four core clinical components: (1) unintentional weight loss (defined as a self-reported involuntary loss of ≥4.5 kg or ≥5% of baseline body weight within the past year), (2) self-reported fatigue (measured using the standardized diagnostic question: “In the last month, have you frequently felt exhausted or lacked the energy to carry out your daily activities?”), (3) muscle weakness (defined by weak handgrip strength according to the sex-adjusted AWGS-2019 cutoff thresholds), and (4) slow gait speed (operationalized via a timed 4-m comfortable walking test). The original “low physical activity” criterion was intentionally excluded from this frailty index to minimize potential statistical redundancy and mitigate multi-collinearity within the multivariable logistic regression model, where regular physical activity frequency was structured separately as a primary independent variable. Within this modified four-item operational framework, participants were initially evaluated based on the number of positive criteria met.

However, because our descriptive analysis revealed a highly skewed distribution with an extremely low cell count in the strict advanced frailty stratum (only 2.44%, *n* = 6 classified as strictly frail, compared to 45.53%, *n* = 112 as pre-frail), the frail and pre-frail categories were collapsed into a single dichotomous group (“Pre-frail/Frail”, *n* = 118) against the reference group (“Robust”, *n* = 128) in the final multivariable regression analysis. This grouping strategy was statistically mandatory to preserve sufficient statistical power, avoid inflated standard errors, and ensure the computational stability of the adjusted model coefficients.

#### 2.3.5. Sarcopenia Assessment (AWGS-2019 Criteria)

Sarcopenia was diagnosed in strict accordance with the AWGS-2019 consensus criteria [2], which encompass three core domains: muscle mass, muscle strength, and physical performance.

For muscle mass assessment, bioelectrical impedance analysis (BIA) was performed using an Omron HBF-375 device (Omron Healthcare, Kyoto, Japan). To ensure data consistency and validity, examinations were strictly standardized across all sites during morning sessions (08:30–11:30 AM). Participants were instructed to fast for at least 2 h prior to testing, void their bladders immediately before measurement, and avoid strenuous physical exertion for 24 h. The assessment was conducted in a standardized standing posture. Although the Omron HBF-375 is an 8-electrode consumer-to-clinical grade device, multi-segment 8-electrode BIA devices have demonstrated high concordance and strong correlation coefficients (*r* > 0.85) with dual-energy X-ray absorptiometry (DXA) for appendicular skeletal muscle mass estimation in Asian and Thai cohorts [33]. The appendicular skeletal muscle index (ASMI) was calculated as appendicular skeletal muscle mass divided by height squared kg/m^2^. Low muscle mass was defined as <7.0 kg/m^2^ for men and <5.7 kg/m^2^ for women [2].

Muscle strength was evaluated using a calibrated digital handgrip dynamometer (Takei Scientific Instruments, Niigata, Japan). Participants performed three maximum-effort trials with each hand while maintaining a standardized standing position (or seated if unable to stand safely) with the elbow fully extended at 180°. Testing alternated between hands to provide a minimum rest period of 60 s between contractions on the same side, and the single highest recorded value from the dominant hand was utilized. Low muscle strength was defined as <28 kg for men and <18 kg for women [2].

Physical performance was assessed using two functional tests: the gait speed test and the Five-Times Sit-to-Stand Test (FTSST). Gait speed was measured using a standardized 6-m course on a flat, unobstructed surface, incorporating a standing start from the 0-m mark. To eliminate acceleration and deceleration artifacts, timing was recorded using a digital stopwatch as the participant walked at their usual comfortable pace between the 1-m mark and the 5-m mark, capturing a standardized 4-m intermediate zone. Low performance was defined as a walking speed < 1.0 m/s [2]. For the FTSST, participants were seated on a standard armless chair (43 cm seat height) with their feet flat on the floor and arms crossed securely over their chest. They were explicitly instructed to stand up completely and sit back down as fast as possible five times continuously without stopping, while keeping their arms crossed. In accordance with AWGS-2019 guidelines, no arm support or pushing off the thighs was permitted, and trials utilizing arm support were deemed invalid. Low performance on the FTSST was defined as an execution time ≥ 12 s [2]. All measurements and diagnostic classifications followed standardized protocols to ensure high data reliability and comparability.

Based on the multi-domain framework of the AWGS-2019 guidelines, participants were progressively staged into three severity categories: (1) possible sarcopenia, defined as low muscle strength or low physical performance with normal muscle mass; (2) confirmed sarcopenia, defined as low muscle mass plus either low muscle strength or low physical performance; and (3) severe sarcopenia, defined as the concurrent presence of deficits across all three domains (low muscle mass, low muscle strength, and low physical performance). All measurements and diagnostic classifications followed these standardized protocols to ensure high data reliability and comparability.

### 2.4. Ethical Considerations

The study was conducted in strict accordance with the principles of the Declaration of Helsinki. The study protocol received formal approval from the Human Research Ethics Committee of Walailak University, Nakhon Si Thammarat, Thailand (Approval No. WUEC-25-312-01; approved on 27 August 2025). Before enrollment, all potential participants received a comprehensive oral and written explanation regarding the study’s objectives, confidentiality measures, procedures, and potential risks. Written informed consent was voluntarily signed and obtained from each participant prior to the initiation of any data collection or physical performance testing. Participants were explicitly informed of their right to withdraw from the study at any time without penalty or loss of healthcare benefits. Although this observational study was not pre-registered in a public clinical trial registry, all procedures strictly adhered to the approved protocol and were reported in compliance with the STROBE (Strengthening the Reporting of Observational Studies in Epidemiology) guidelines [34]. To protect participant confidentiality, all personal identifiers were removed, and the dataset was fully anonymized and used exclusively for research purposes.

### 2.5. Statistical Analyses

All data underwent a thorough review for completeness and internal consistency prior to formal analysis. Our field data collection utilized an immediate on-site quality-control protocol, resulting in zero missing values (*n* = 0) across all parameters and eliminating the need for statistical imputation. Statistical analyses were conducted using SPSS software version 22.0 (IBM Corp., Armonk, NY, USA). Descriptive statistics were utilized to summarize the baseline characteristics, reporting frequencies and percentages for categorical variables, and means with standard deviations (SD) for continuous variables. Sex-specific crude prevalence rates of sarcopenia were calculated along with their corresponding 95% confidence intervals (CIs). Comparisons of baseline characteristics and potential risk factors between the sarcopenia and non-sarcopenia groups were performed using Pearson’s Chi-square tests (x^2^), or Fisher’s exact tests for categorical variables where expected cell counts were fewer than five, and independent sample *t*-tests for continuous variables.

A multivariable logistic regression model was developed to identify independent factors associated with sarcopenia. For this analysis, the dependent variable was operationalized as a binary outcome: Confirmed Sarcopenia (coded as 1) versus non-sarcopenia (coded as 0, which encompassed both robust individuals and those with possible sarcopenia). The final model was constructed using a manual backward elimination procedure. Initially, candidate variables were selected for multivariable entry if they demonstrated a *p*-value < 0.20 in the crude univariate analyses, or if they possessed established epidemiological relevance in the literature. Given their well-established biological association with muscle degradation, age and sex were forced and retained in the multivariable model a priori as structural covariates. Furthermore, to adjust for potential spatial clustering across the multi-site sampling framework, district was also entered as a fixed block structural covariate. During the backward elimination phase, non-significant candidate variables with the lowest statistical contribution were removed one-by-one. Variables were strictly retained in the definitive model if their independent significance maintained a *p*-value < 0.05, or if their removal altered the primary exposure odds ratios (ORs) by more than 10%, indicating active confounding [35,36].

All continuous independent variables evaluated within the regression framework (such as age and BMI) were verified for the assumption of linearity in the logit using the Box-Tidwell transformation test. All tested transformations yielded non-significant interaction terms (*p* > 0.05), confirming that the assumption of linearity was met. To enhance statistical power and precision, age and BMI were treated as continuous variables and reported per 5-year and per 1 kg/m^2^ increment, respectively. Multicollinearity among the independent variables was evaluated using the variance inflation factor (VIF), with a threshold of <5.0 indicating the absence of severe collinearity. The goodness-of-fit of the final regression model was verified using the Hosmer–Lemeshow test. Adjusted odds ratios (AORs) with their corresponding 95% CIs were calculated. Because this was an exploratory risk-factor modeling framework testing a predefined, theoretically driven set of distinct covariates, adjustments for multiple comparisons were not applied to the regression coefficients to avoid inflating Type II error rates; however, for all post hoc pairwise comparisons between individual geographic districts, a strict Bonferroni adjustment was applied. For all statistical tests, significance was defined as a two-tailed *p*-value < 0.05.

## 3. Results

### 3.1. Participant Characteristics and Health Status

From the initial sampling frame of 450 registered older adults, 140 individuals who did not attend the monthly health promotion meetings were not approached. Of the 310 individuals actively approached, 42 were excluded based on pre-defined clinical health criteria and 22 declined to participate, resulting in a final sample of 246 community-dwelling older adults who completed all protocols (participation rate = 79.4%). The sample was predominantly female (88.62%), with a mean age of 69.4 ± 7.1 years (age range: 60–92 years). Comprehensive sociodemographic profiles and health characteristics of the entire cohort are summarized in Table 1. To contextualize the economic status, a monthly income threshold of 5000 Thai Baht (THB) (approximately 145 United States Dollars [USD]) was used to classify the lower-income group. This baseline represents a socioeconomically vulnerable segment within Nakhon Si Thammarat, falling between the national poverty line (approximately 2803–3000 THB/month) and the provincial minimum wage baseline (approximately 8400 THB/month).

To provide a comprehensive local geographic context, baseline sociodemographic profiles, health behaviors, and clinical parameters were stratified across the three studied districts: Tha Sala (*n* = 80), Pak Phanang (*n* = 80), and Ron Phibun (*n* = 86), as detailed in Table 2. There were no statistically significant differences across the districts regarding sex distribution (*p* = 0.942), age groups (p=0.941), or baseline BMI categories (*p* = 0.218). However, significant geographic variations were observed in educational attainment (*p* = 0.048) and monthly income (*p* = 0.026). Specifically, participants from Pak Phanang District demonstrated a higher proportion of primary education or lower (75.00%) and a greater percentage of lower-income individuals earning < 5000 THB (66.25%) compared to those living in Tha Sala and Ron Phibun. Notably, physical activity levels varied substantially across the three districts (*p* < 0.001), while the overall prevalence of sarcopenia and its individual diagnostic components did not differ significantly by geographic location (*p* > 0.05). (See Supplementary Material for details).

### 3.2. Prevalence of Sarcopenia and Nutritional Status

Following the AWGS-2019 criteria, the overall crude prevalence of sarcopenia in the study population was 27.64% (95% CI: 22.14–33.72%; (*n* = 68)). When stratified by sex, the prevalence of sarcopenia exhibited a substantial divergence: among female participants, the prevalence was 20.18% (95% CI: 15.06–26.15%; (*n* = 44)), whereas among male participants, the prevalence reached 85.71% (95% CI: 67.33–95.97%; (*n* = 24)). The wide 95% confidence interval observed within the male subgroup explicitly demonstrates the statistical instability of this specific stratum estimate, which is constrained by the small male sample size (*n* = 28). Analysis of the individual sarcopenia components revealed that 43.09% of participants exhibited low muscle mass, with a mean appendicular skeletal muscle mass index (ASMI) of 5.80 ± 1.10 kg/m^2^. Low muscle strength was identified in 30.08% of the cohort (mean handgrip strength: 22.45 ± 5.42 kg). Furthermore, more than half of the participants (51.62%) demonstrated reduced physical performance, as evidenced by a mean five-times sit-to-stand time of 11.42 ± 3.15 s and a mean gait speed of 0.98 ± 0.21 m/s. Regarding nutritional status, the MNA-SF assessment showed that while 46.34% of participants maintained a normal status, more than half were at nutritional risk; specifically, 50.41% were classified as at risk of malnutrition, and 3.25% were identified as malnourished (Table 3).

### 3.3. Factors Associated with Sarcopenia

Univariate analysis using Pearson’s Chi-square tests revealed that several baseline factors were significantly associated with sarcopenia, including age group ($\chi^{2}=58.62$), physical activity ($\chi^{2}=48.60$), nutritional status ($\chi^{2}=49.82$), and frailty status ($\chi^{2}=42.18$; all *p* < 0.001).

#### 3.3.1. Multivariable Analysis (Risk and Protective Factors)

In the multivariable logistic regression model, several independent factors remained significantly associated with sarcopenia after adjusting for potential confounders (Table 4). Older adults aged ≥ 80 years exhibited a fourfold increase in sarcopenia risk compared to those aged 60–79 years (AOR = 4.25, 95% CI: 1.12–16.14; *p* < 0.033). Corroborating this, the continuous analysis model demonstrated that each 5-year increase in age was associated with a 52% higher likelihood of sarcopenia (AOR = 1.52, 95% CI: 1.14–2.03; p=0.005. Conversely, higher educational attainment served as a significant protective factor, with those achieving a secondary education or higher showing an 88% reduction in the odds of sarcopenia (AOR = 0.12, 95% CI: 0.02–0.78; p=0.025).

Sex was not identified as an independent factor in the final adjusted model (AOR = 0.45, 95% CI: 0.14–1.42; *p* = 0.172). In response to the reviewer’s recommendation regarding the low proportion of male participants (11.38%, *n* = 28), a detailed descriptive analysis was conducted using the individual AWGS-2019 diagnostic criteria. Among male participants, 85.71% (*n* = 24) exhibited a concurrent presentation of low appendicular skeletal muscle mass index (ASMI < 7.0 kg/m^2^), reduced muscle strength (handgrip strength < 28 kg), and diminished physical performance (gait speed < 1.0 m/s or FTSST ≥ 12 s). As a result, severe sarcopenia was identified in 85.71% (*n* = 24) of the men, while only 14.29% (*n* = 4) were classified as non-sarcopenic. This clinical distribution indicates that the high baseline rate of sarcopenia among male participants is attributable to a consistent, multi-domain deficit in muscle mass, strength, and physical function.

#### 3.3.2. Anthropometric and Lifestyle Factors

Compared to the normal BMI range (18.50–22.90 ${\mathrm{kg}/m}^{2}$), underweight participants ($18.50{\;\mathrm{kg}/m}^{2}$) had significantly higher odds of developing sarcopenia (AOR = 3.58, 95% CI: 1.20–10.65; p=0.022). To increase statistical power and clinical relevance, the overweight and obese categories were collapsed into a single group ($\geq 23.00{\;\mathrm{kg}/m}^{2}$), which demonstrated an 81% reduction in the odds of sarcopenia (AOR = 0.19, 95% CI: 0.09–0.40; p<0.001). This protective trend was further evidenced in the continuous model, where each $1{\;\mathrm{kg}/m}^{2}$ increase in BMI was associated with a 22% decrease in sarcopenia risk (AOR = 0.78, 95% CI: 0.69–0.88; p<0.001). Furthermore, regular physical activity (≥3 times/week) was identified as a key protective factor (AOR = 0.21, 95% CI: 0.08–0.54; p=0.001). However, when physical activity frequency was analyzed as a continuous variable (times/week), the association was not statistically significant (p=0.421), suggesting that a specific frequency threshold is required to achieve protective benefits.

#### 3.3.3. Nutritional and Functional Status

**Figure 2:**
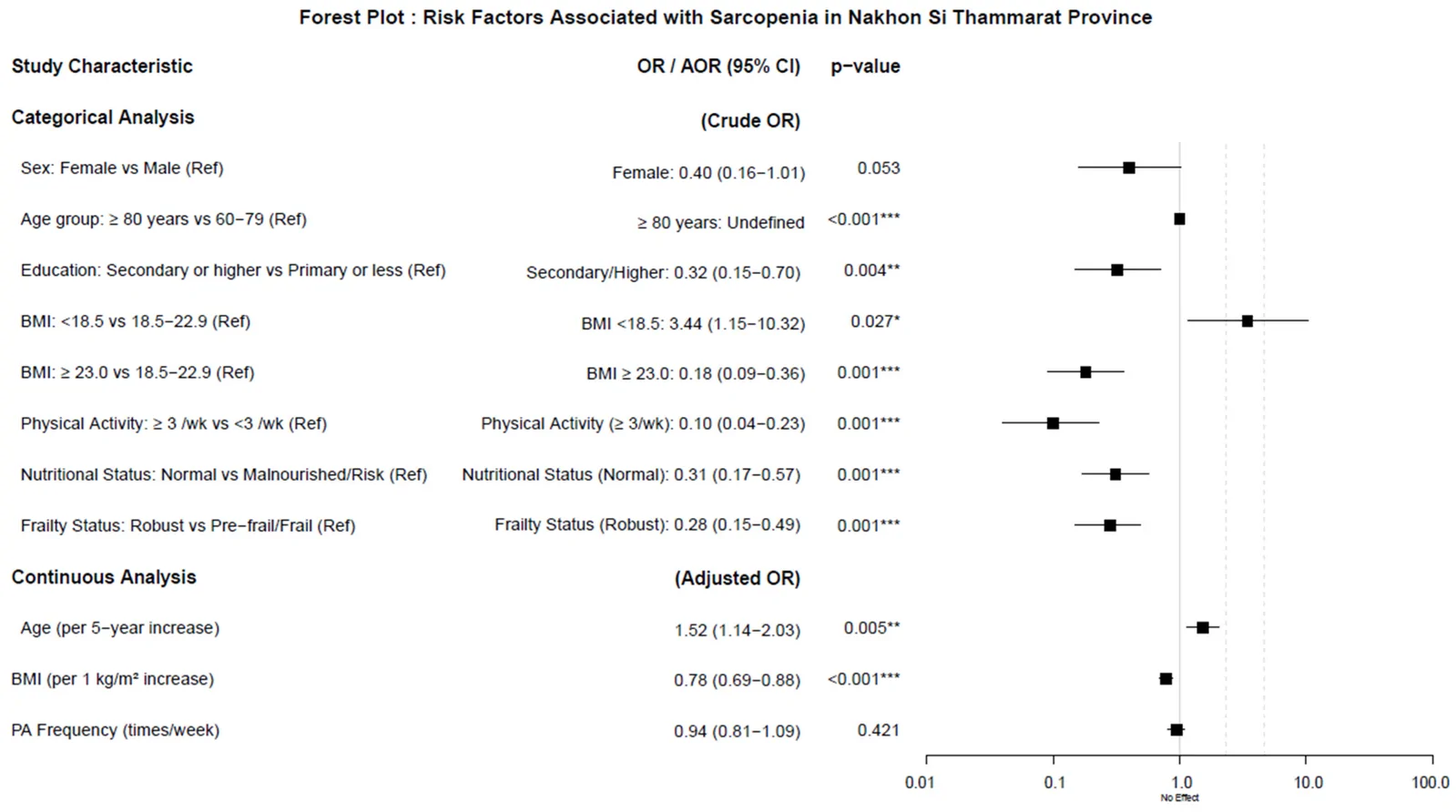
Independent Risk and Protective Factors for Sarcopenia among Community-Dwelling Older Adults: Results of Multivariable Logistic Regression (*n* = 246). Forest plot of adjusted odds ratios (AORs) for independent risk and protective factors associated with sarcopenia. The plot displays results from both categorical analysis (top) and continuous analysis (bottom) Squares and horizontal error bars represent the AORs and 95% confidence intervals (CIs), respectively. The vertical dashed line at 1.0 indicates the null effect. The multivariable model was adjusted for sex, age, education level, BMI, physical activity, nutritional status, and frailty status. * *p* < 0.05, ** *p* < 0.01, *** *p* < 0.001. Note: Ref = Reference group; OR = Crude Odds Ratio; AOR = Adjusted Odds Ratio; CI = Confidence Interval.

Finally, nutritional and functional status played critical roles in relation to sarcopenia. A normal nutritional status was associated with 76% lower odds of having sarcopenia (AOR = 0.24, 95% CI: 0.08–0.71; p=0.010). Regarding frailty, while robust individuals appeared to have lower odds compared to the pre-frail/frail group (AOR = 0.40, 95% CI: 0.15–1.08), this association did not reach statistical significance in the final adjusted model (p=0.068). The detailed results of the univariate and multivariable analyses are presented in Table 4, and the independent factors are visually summarized in the forest plot (Figure 2).

## 4. Discussion

The overall crude prevalence of sarcopenia observed in this cohort was substantial. When evaluated against domestic literature utilizing the standardized AWGS-2019 criteria, our observed prevalence is notably higher than that reported by Sri-On et al. [22], who identified a lower prevalence of confirmed sarcopenia among emergency-department-discharged older patients and general community-dwelling seniors in more urbanized regions of central Thailand. On a broader regional scale, our estimates significantly outpace reported prevalence rates from community-dwelling populations in highly developed East Asian nations such as Japan and South Korea, which conventionally range between 8% and 15% [37,38]. Conversely, our findings show a closer structural alignment with specific socioeconomically vulnerable or urban-fringe cohorts investigated in Singapore and mainland China [39]. These geographical and epidemiological variations in prevalence can be explained by several interconnected factors, including our explicit focus on a rural, lower-income population, a higher baseline representation of advanced age cohorts within our sampling framework, and profound regional disparities in lifetime occupational trajectories and dietary protein access unique to southern Thai communities. This substantial prevalence underscores a critical public health burden in rural Southern Thai communities, where access to specialized geriatric care is often structurally constrained. Furthermore, the high proportion of participants demonstrating low physical performance indicates that a major segment of this population resides in the early or “possible sarcopenia” stage, highlighting an urgent need to integrate routine screening and community-based primary care interventions, such as resistance training and structured nutritional counseling, to mitigate progression toward severe disability and loss of functional independence [2,40].

Advanced age was identified as a primary determinant of sarcopenia in this cohort, validating established geriatric literature [7,15]. Participants in the oldest-old age stratum exhibited a markedly elevated risk of sarcopenia compared to their younger counterparts. In the univariate analysis, the oldest-old subgroup demonstrated a significant association with sarcopenia status via Fisher’s exact test. Although the small sample size within this advanced age stratum resulted in lower precision, the substantial risk is physiologically attributable to cumulative age-related catabolism. These include compromised muscle protein synthesis, impaired neuromuscular recruitment, and chronic low-grade systemic inflammation, frequently referred to as “inflammaging” [41,42]. Corroborating this, our continuous model demonstrated that incremental increases in age were independently associated with a higher likelihood of sarcopenia, reinforcing chronological aging as a robust risk factor that exhibits a distinct acceleration in the eighth decade of life [42,43]. In rural Southern Thailand, these biological shifts are further compounded by environmental and lifestyle constraints, as the oldest-old are highly susceptible to reduced mobility and age-related anorexia, leading to inadequate macronutrient and micronutrient intake [15,42,43,44]. To counter this non-linear risk expansion, we recommend age-stratified clinical approaches: universal, proactive field screening utilizing the SARC-F tool or calf-circumference measurements should be mobilized at the primary care level starting at the age of 60, transitioning to mandatory objective functional testing (handgrip strength and five-times sit-to-stand tests) for older individuals to optimize early physical therapy routing.

A key epidemiological finding that warrants careful methodological evaluation is the pronounced gender divergence observed in our descriptive analysis, where the crude prevalence of sarcopenia was notably higher among males than females. This stark contrast may reflect localized gender-specific occupational histories in rural Southern Thailand, where women traditionally engage in prolonged, low-to-moderate intensity agricultural tasks—such as rubber tapping and small-scale subsistence farming—across their life course. These sustained labor patterns may act as a continuous stimulus for physical conditioning and musculoskeletal mass retention, buffering females against accelerated post-retirement sarcopenic declines. However, in alignment with the reviewer’s constructive assessment, this high male prevalence estimate must be interpreted with extreme caution, as the small absolute male sample size severely limits the validity and generalizability of these gender-specific interpretations. The non-probability convenience sampling framework resulted in a pronounced gender imbalance, restricting the statistical power of sex-specific sub-analyses. Consequently, when sex was forced into the multivariable logistic regression model as a baseline structural covariate, no statistically significant independent association was observed. This implies that the apparent high crude prevalence among men is likely a statistical artifact driven by an underpowered sample size (Type II error) rather than a definitive biological predisposition unique to rural Southern Thai males. Accordingly, any statistical inferences drawn from such heavily restricted subgroups within this cross-sectional design must be interpreted with strict caution, as they are highly susceptible to selection artifacts like the healthy volunteer effect or index event bias [43]. To overcome this limitation and truly validate the impact of career labor on muscle aging, future studies should move away from speculative occupational proxies and instead integrate objective, standardized measurement tools, such as cumulative lifetime physical workload indices or job exposure matrices (JEM) [45], paired with a larger, gender-balanced recruitment framework.

Nevertheless, from a clinical phenotyping perspective, our AWGS-2019 multi-domain diagnostic protocol revealed that sarcopenic male participants exhibited consistent, simultaneous deficits across all three diagnostic domains: appendicular skeletal muscle mass, handgrip strength, and physical performance. While this uniform presentation implies severe musculoskeletal degradation among the specific subgroup of older men who actively attended our community health centers, the convenience recruitment design severely limits the generalizability of these high-risk profiles to the broader male population of Southern Thailand, necessitating a cautious and conservative interpretation of these cross-sectional parameters. Structurally, older men in rural Thai communities frequently engage in prolonged daytime agricultural labor or rubber tapping away from village centers, making them less predisposed to attend senior clubs or community health screenings compared to older women. Future studies should deploy targeted home-based health assessments and implement sex-stratified random sampling from municipal population registries to secure an equitable demographic representation and validate sex-specific sarcopenia dynamics.

Socioeconomic status emerged as a significant structural determinant of muscle health in this population. Participants achieving educational attainment beyond the primary level exhibited substantially lower odds of developing sarcopenia. This protective association is mediated by a multi-step pathway where higher education enhances comprehensive health literacy. This increased literacy directly facilitates the adoption of high-quality nutritional habits, greater adherence to health-promoting and preventive physical behaviors, and early compliance with specialized exercise protocols. Individuals with higher education levels are typically more cognizant of the importance of nutritional density and structured physical activity, both of which are essential for preserving muscle mass and mitigating neuromuscular decay in later life [46]. Correspondingly, a lower monthly income was significantly associated with an increased likelihood of sarcopenia in the univariate analysis. This highlights the role of income as a vital macro-structural determinant; a monthly income below 5000 THB acts as a direct financial bottleneck that severely restricts an older individual’s purchasing power for high-yield, premium macronutrients—such as lean animal proteins, fresh produce, and nutritional supplements—while creating a material barrier to accessing specialized preventive healthcare and fitness resources [47,48]. In rural Southern Thailand, where socioeconomic stability fluctuates, these findings emphasize that sarcopenia is not merely a biological consequence of aging but is heavily shaped by structural socioeconomic disadvantages. To address these socioeconomic disparities, specific public health policy interventions must be mobilized through the Ministry of Public Health. We advocate for the establishment of targeted community-level programs that distribute subsidized, culturally acceptable protein alternatives and fund structured progressive resistance training sessions. These resources should be managed and executed by local public health officers and village health volunteers (VHVs) to ensure they directly reach economically disadvantaged and vulnerable older adults within lower-income sub-districts [49].

Body Mass Index (BMI) was identified as a powerful independent protective factor against sarcopenia in this cohort. Enrollees with a higher BMI experienced a substantially lower risk of sarcopenia compared to those within the normal range. Continuous analysis corroborated this robust relationship, showing that incremental increases in BMI were consistently associated with a reduced risk of sarcopenia. This notable protective trend aligns with the “obesity paradox” frequently described in geriatric research, wherein an elevated body mass index—traditionally considered a metabolic risk factor in younger cohorts—is associated with superior musculoskeletal outcomes and reduced mortality in later life [46,47]. Physiologically, a higher BMI may signify superior energy and structural reserves, acting as an anabolic buffer against age-related hypercatabolism. Conversely, a low BMI typically reflects chronic energy deficiency and malnutrition, which induce anabolic resistance and accelerate muscle wasting [48,49]. However, this protective trend must be evaluated with strict caution due to potential residual confounding and the complete absence of regional tissue-specific body composition data within basic anthropometric screenings. Because BMI is a surrogate metric that does not differentiate between adipose tissue and skeletal muscle mass, a higher BMI may simply indicate a greater reserve of lean mass rather than excess adiposity. It remains critical to distinguish between the generalized mechanical cushioning effects of stable body mass and the highly hazardous phenotype of sarcopenic obesity. In this pathogenic condition, high ectopic and visceral adiposity actively masks underlying severe muscle depletion while inducing a synergistic cascade of chronic low-grade systemic inflammation, metabolic syndrome, and physical disability [50,51]. Because this study employed BMI without concurrent body fat percentage adjustments, the precise physiological contribution of fat versus lean mass remains undetermined, warranting further validation via bioelectrical impedance analysis (BIA) or dual-energy X-ray absorptiometry (DXA) [2]. Consequently, clinical practice guidelines for older adults in rural Southern Thailand should avoid endorsing ambiguous weight gain; instead, they must prioritize maintaining a stable weight across the later life course while actively preserving or building lean muscle mass over traditional weight-loss objectives [46,49].

Regular physical activity was confirmed as an essential non-pharmacological protective factor against sarcopenia. Participants exercising three or more times per week exhibited substantially lower odds of sarcopenia compared to less active individuals. Interestingly, while the categorical analysis demonstrated powerful statistical significance, the continuous analysis of exercise frequency did not reach statistical significance. This discrepancy clearly indicates a threshold effect, suggesting that protective benefits for skeletal muscle are unlocked only when a minimum frequency threshold (at least three times per week) is consistently maintained, rather than through low-frequency incremental changes [49]. Regular physical activity counteracts anabolic resistance and promotes myofibrillar protein synthesis [46]. However, this threshold finding must be interpreted carefully within the context of measurement limitations. Because physical activity was captured exclusively via self-reported frequency without objective metabolic equivalent task (MET)-minutes or accelerometer verification, a degree of misclassification bias might be present. We transparently acknowledge the lack of objective intensity and duration data as a limitation. In agrarian rural Southern Thai communities, where manual occupational labor (e.g., agricultural tasks or rubber tapping) is inherently intensive, individuals with low leisure exercise but high occupational exertion may have been misclassified into the less active reference stratum. This unmeasured variance in physical activity intensity likely introduced a non-differential misclassification, thereby biasing our observed continuous effect size toward the null and underestimating the true protective capacity of absolute physical exertion. Regarding clinical exercise prescription for sarcopenia prevention in this setting, it is vital to specify the type of training. While general aerobic activities (such as brisk walking) promote cardiorespiratory fitness and systemic metabolic health, targeted progressive resistance training (PRT)—using accessible options like bodyweight exercises, elastic resistance bands, or free weights—must be prioritized as the clinical gold standard to directly trigger myofibrillar hypertrophy, enhance neuromuscular activation, and arrest functional mobility decline.

Furthermore, nutritional status, assessed via the MNA-SF, served as a robust independent protective factor, with normal nutrition strongly correlating with a reduced likelihood of sarcopenia. This underscores that dietary quality and nutrient density, rather than simple meal frequency, act as primary drivers of muscle health [52,53]. In rural Southern Thai contexts, where traditional diets are heavily carbohydrate-dominant and structurally lack high-bioavailability proteins, this distinction is crucial [54,55]. Low consumption of dairy products and essential amino acids like leucine compromises the muscle protein synthesis stimulus [55,56]. Nevertheless, since the rapid screening nature of the MNA-SF leaves precise daily protein intake unquantified, residual confounding from dietary quality cannot be entirely ruled out. This under-measurement likely diluted the observable nuances of specific nutrient depletion and anabolic resistance thresholds, meaning the definitive protective association of a targeted high-protein profile on muscle preservation is potentially stronger than reported here. To translate these findings into precise nutritional guidelines, dietary recommendations must go beyond general advice and provide concrete quantitative targets. In alignment with international clinical nutrition consensus statements, older adults diagnosed with sarcopenia or identified as being at nutritional risk should maintain a targeted daily intake of 1.2 to 1.5 g of high-quality protein per kilogram of body weight per day (g/kg/day). Public health strategies in Southern Thailand must emphasize protein-dense nutrition by leveraging economically accessible and culturally appropriate local resources. These include fresh coastal marine fish (e.g., mackerel), local poultry, eggs, legumes, and fermented or soy-based products (such as tofu), which bypass financial constraints while effectively supplying essential amino acids like leucine to stimulate muscle synthesis [57].

In addition to macronutrient optimization, the critical role of key micronutrients—specifically Vitamin D and Calcium—must be systematically addressed to ensure structural muscle integrity. Suboptimal levels of Vitamin D undermine musculoskeletal wellness by reducing myofibrillar cross-bridge cycling and weakening neuromuscular coordination, whereas concurrent Calcium deficiencies accelerate bone turnover and exacerbate overall age-related physical frailty, necessitating targeted micro-nutritional screening and community-level supplementation protocols in primary care settings [58,59].

### 4.1. Study Limitations

Our study possesses several structural limitations that must be prioritized when interpreting the findings. First, the cross-sectional design inherently measures exposure variables and sarcopenic phenotypes concurrently, which prevents the establishment of temporal causality. Consequently, all identified socio-demographic, anthropometric, and behavioral factors must be viewed strictly as statistical associations rather than proven causal relationships.

Second, a pronounced gender imbalance was present within our cohort, with female participants comprising 88.62% of the sample. This severe skew severely limits the statistical power of sex-specific sub-analyses and may introduce a non-differential underestimation of the absolute population sarcopenia rate, given that older males in rural communities often undergo distinct, sharper muscle mass loss patterns post-retirement but were underrepresented here due to their lower tendency to attend community health centers during daytime hours.

Third, our field-based anthropometric measurement relied on bioelectrical impedance analysis (BIA) rather than a laboratory-grade dual-energy X-ray absorptiometry (DXA) reference standard. While BIA is highly feasible and validated for large-scale rural screening, it is susceptible to minor non-differential misclassification errors driven by participant hydration status and ambient temperature. This technical constraint typically biases unadjusted continuous associations toward the null, thereby potentially underestimating the true magnitude of the effect sizes reported in our regression models.

Additionally, the study is vulnerable to selection bias through the “healthy volunteer effect”. Because our recruitment framework relied on ambulatory individuals capable of independently traveling to and attending community health promotion meetings, those with advanced frailty, severe mobility impairments, or institutionalized status were likely omitted. This selective exclusion means our findings may under-represent the true clinical burden and severe stages of sarcopenia in the broader population.

Finally, while the Mini Nutritional Assessment-Short Form (MNA-SF) serves as a highly valid and rapid field screening tool, it depends partly on subjective self-reporting for specific parameters, such as recent acute psychological stress or subjective dietary intake decline over the past three months. This reliance may introduce mild recall or social desirability bias compared to objective, multi-day biochemical biomarker tracking or detailed dietary logs, which should be integrated into future longitudinal trials to further minimize residual confounding.

### 4.2. Future Research Directions

To address these limitations, future prospective investigations should transition from rapid screening phenotypes toward comprehensive, multi-modal diagnostic approaches. Subsequent studies should utilize sex-stratified random sampling designs from municipal registries and deploy active home-based screening strategies during evening hours to secure equitable demographic representation and validate these findings across genders. Furthermore, future research should integrate detailed 24-h dietary recalls or weighted food records to precisely quantify macronutrient and protein intake density relative to anabolic resistance thresholds. Incorporating objective serum biochemical markers—such as pre-albumin, transferrin, total iron-binding capacity, and absolute lymphocyte counts—will be paramount to establishing a precise clinical diagnosis of systemic malnutrition. To achieve objective physical activity profiling, future designs should utilize accelerometer-based monitoring alongside the International Physical Activity Questionnaire (IPAQ) [60] to record exact physical activity intensity (MET-minutes) and distinguish between resistance and aerobic modalities. Finally, researchers should capture precise clinical and socioeconomic dimensions by deploying validated indices to quantify comorbidity burden (e.g., the Charlson Comorbidity Index) [61], tracking detailed long-term medication profiles (specifically statins, corticosteroids, and beta-blockers) [62], and expanding socioeconomic parameters to incorporate household wealth indices and lifetime occupational exposure histories. Integrating these comprehensive metrics will elucidate the definitive longitudinal, bi-directional pathways connecting clinical, nutritional, and behavioral depletion with muscular degradation in older adults.

## 5. Conclusions

In conclusion, this study demonstrates a substantial burden of sarcopenia among community-dwelling older adults in rural Southern Thailand, with a crude prevalence of 27.64% and over half of the cohort residing in the “possible sarcopenia” stage. Within this cross-sectional framework, advanced chronological age (≥75 years), underweight status, lower socioeconomic position (limited education and low family income), low nutritional status (MNA-SF screening), and a lack of frequent physical activity (≤3 times/week) were found to be significantly associated with an increased likelihood of sarcopenic phenotypes. To effectively address these public health and clinical vulnerabilities, community health workers, local nursing officers, and village health volunteers (VHVs) should systematically screen older adults within primary healthcare networks using a combined protocol of the MNA-SF and the AWGS-2019 criteria. Identifying at-risk or early-stage individuals will allow for the targeted implementation of community-level interventions, specifically customized progressive resistance training (PRT) and culturally appropriate, protein-dense nutritional counseling, to maintain optimal musculoskeletal health across the later life course. Finally, because the inherent properties of our cross-sectional design preclude any definitive causal inferences regarding directionality or temporal mechanisms, large-scale longitudinal cohort studies and prospective randomized controlled trials are urgently required to establish the precise causal relationships between localized socioeconomic structural barriers, long-term dietary exposures, and the progression of sarcopenia in this rural sub-population.

## Figures and Tables

**Table 1:** Sociodemographic and health characteristics of participants (*n* = 246).

Characteristics		Number	Percentage
Sex			
	Male	28	11.38
	Female	218	88.62
Age (years)			
	60–69	132	53.65
	70–79	96	39.02
	≥80	18	7.31
Education level			
	Primary education or lower	168	68.29
	Secondary education or higher	78	31.71
Monthly income			
	<5000 THB	142	57.72
	≥5000 THB	104	42.28
Health status			
	Presence of chronic conditions	198	80.48
	Regular medication use	151	61.38
	History of falls (past year)	66	26.82

**Table 2:** Sociodemographic characteristics, health profiles, and clinical components stratified by district (*n* = 246).

Characteristics	Total (*n* = 246)	Tha Sala District (*n =* 80)	Pak Phanang District (*n* = 80)	Ron Phibun District (*n* = 86)	*p*-Value
Categorical Analysis					
Sex					0.942 ^b^
Male	28 (11.38%)	9 (11.25%)	9 (11.25%)	10 (11.63%)	
Female	218 (88.62%)	71 (88.75%)	71 (88.75%)	76 (88.37%)	
Age group (years)					0.941 ^b^
60–79	228 (92.68%)	74 (92.50%)	74 (92.50%)	80 (93.02%)	
≥80	18 (7.31%)	6 (7.50%)	6 (7.50%)	6 (6.98%)	
Education level					0.048 ^b^
Primary or less	168 (68.29%)	50 (62.50%)	60 (75.00%)	58 (67.44%)	
Secondary or higher	78 (31.71%)	30 (37.50%)	20 (25.00%)	28 (32.56%)	
Monthly income					0.026 ^b^
<5000 THB	142 (57.72%)	41 (51.25%)	53 (66.25%)	48 (55.81%)	
≥5000 THB	104 (42.28%)	39 (48.75%)	27 (33.75%)	38 (44.19%)	
Body mass index (kg/m^2^)					0.218 ^b^
<18.50	22 (8.94%)	7 (8.75%)	8 (10.00%)	7 (8.14%)	
18.50–22.90	78 (31.71%)	25 (31.25%)	24 (30.00%)	29 (33.72%)	
≥23.0	146 (59.35%)	48 (60.00%)	48 (60.00%)	50 (58.14%)	
Physical activity					<0.001 ^b^
<3 times/week	138 (56.10%)	45 (56.25%)	47 (58.75%)	46 (53.49%)	
≥3 times/week	108 (43.90%)	35 (43.75%)	33 (41.25%)	40 (46.51%)	
Nutritional status					0.241 ^b^
Malnourished/Risk	132 (53.66%)	40 (50.00%)	47 (58.75%)	45 (52.33%)	
Normal	114 (46.34%)	40 (50.00%)	33 (41.25%)	41 (47.67%)	
Frailty status					0.184 ^b^
Pre-frail/Frail	118 (47.97%)	37 (46.25%)	40 (50.00%)	41 (47.67%)	
Robust	128 (52.03%)	43 (53.75%)	40 (50.00%)	45 (52.33%)	
Health profiles & Sarcopenia components					
Presence of chronic conditions, *n* (%)	198 (80.48%)	64 (80.00%)	65 (81.25%)	69 (80.23%)	0.982 ^b^
Regular medication use, *n* (%)	151 (61.38%)	48 (60.00%)	50 (62.50%)	53 (61.63%)	0.938 ^b^
History of falls (past year), *n* (%)	66 (26.82%)	21 (26.25%)	23 (28.75%)	22 (25.58%)	0.912 ^b^
Low muscle strength, *n* (%)	74 (30.08%)	22 (27.50%)	26 (32.50%)	26 (30.23%)	0.782 ^b^
Low physical performance, *n* (%)	127 (51.62%)	38 (47.50%)	41 (51.25%)	48 (55.81%)	0.542 ^b^
Low muscle mass, *n* (%)	106 (43.09%)	31 (38.75%)	38 (47.50%)	37 (43.02%)	0.528 ^b^
Sarcopenia, *n* (%)	68 (27.64%)	20 (25.00%)	25 (31.25%)	23 (26.74%)	0.672 ^b^
Non-sarcopenia, *n* (%)	178 (72.36%)	60 (75.00%)	55 (68.75%)	63 (73.26%)	
Continuous Analysis					
Age (per 5-year increase), Mean ± SD	69.40 ± 7.10	69.20 ± 6.80	69.90 ± 7.50	69.10 ± 7.00	0.825 ^a^
BMI (per 1 kg/m^2^ increase), Mean ± SD	23.85 ± 3.65	23.90 ± 3.52	23.65 ± 3.80	23.98 ± 3.62	0.841 ^a^
PA frequency (times/week), Mean ± SD	2.85 ± 1.42	2.80 ± 1.35	2.75 ± 1.48	2.98 ± 1.40	0.546 ^a^
Handgrip strength (kg), Mean ± SD	22.45 ± 5.42	22.90 ± 5.18	21.95 ± 5.52	22.48 ± 5.58	0.482 ^a^
Five-times sit-to-stand (s), Mean ± SD	11.42 ± 3.15	11.22 ± 2.92	11.58 ± 3.22	11.46 ± 3.32	0.724 ^a^
Gait speed (m/s), Mean ± SD	0.98 ± 0.21	1.00 ± 0.19	0.96 ± 0.22	0.97 ± 0.21	0.415 ^a^
ASMI (kg/m^2^), Mean ± SD	5.80 ± 1.10	5.90 ± 1.00	5.70 ± 1.20	5.80 ± 1.10	0.512 ^a^

BMI = Body Mass Index; PA = Physical Activity; ASMI = Appendicular Skeletal Muscle Mass Index; SD = Standard Deviation. ^a^ Calculated via One-way ANOVA. ^b^ Calculated via Pearson’s Chi-square test.

**Table 3:** Nutritional status and sarcopenia components among participants (*n* = 246).

Parameters	Classification	*n* (%)	Mean ± SD
Nutritional status (MNA-SF)	Normal	114 (46.34)	-
	At risk of malnutrition	124 (50.41)	-
	Malnourished	8 (3.25)	-
Sarcopenia components	Low muscle strength	74 (30.08)	-
	Low physical performance	127 (51.62)	-
	Low muscle mass	106 (43.09)	-
Overall sarcopenia (AWGS-2019)	Sarcopenia	68 (27.64)	-
	Non-sarcopenia	178 (72.36)	-
Clinical measures	Handgrip strength (kg)	-	22.45 ± 5.42
	Appendicular skeletal muscle mass index (kg/m^2^)	-	5.80 ± 1.10
	Five-times sit-to-stand (seconds)	-	11.42 ± 3.15
	Gait speed (m/s)	-	0.98 ± 0.21

MNA-SF = Mini Nutritional Assessment–Short Form; AWGS-2019 = Asian Working Group for Sarcopenia 2019; SD = Standard Deviation.

**Table 4:** Risk factors associated with sarcopenia among participants in Nakhon Si Thammarat Province.

Characteristics		Sarcopenia (*n*)	Non-Sarcopenia (*n*)	Crude OR (95% CI)	*p*-Value	AOR * (95% CI)	*p*-Value
Categorical Analysis							
Sex							
	Male	24	4	1	Ref.	1	Ref.
	Female	44	174	0.40 (0.16–1.01)	0.053	0.45 (0.14–1.42)	0.172
Age group (years)							
	60–79	50	178	1	Ref.	1	Ref.
	≥80	18	0	Undefined ^c^	<0.001 ***^,b^	4.25 (1.12–16.14)	0.033 *
Education level							
	Primary or less	56	112	1	Ref.	1	Ref.
	Secondary or higher	12	66	0.32 (0.15–0.70)	0.004 **^,a^	0.12 (0.02–0.78)	0.025 *
Body mass index (kg/m^2^)							
	<18.5	16	6	3.44 (1.15–10.32)	0.027 *^,a^	3.58 (1.20–10.65)	0.022 *
	18.5–22.9	34	44	1	Ref.	1	Ref.
	≥23.0	18	128	0.18 (0.09–0.36)	<0.001 ***^,a^	0.19 (0.09–0.40)	<0.001 ***
Physical activity							
	<3 times/week	60	78	1	Ref.	1	Ref.
	≥3 times/week	8	100	0.10 (0.04–0.23)	<0.001 ***^,a^	0.21 (0.08–0.54)	0.001 ***
Nutritional status							
	Malnourished/Risk	50	36	1	Ref.	1	Ref.
	Normal	18	142	0.31 (0.17–0.57)	<0.001 ***^,a^	0.24 (0.08–0.71)	0.010 *
Frailty status							
	Pre-frail/Frail	62	56	1	Ref.	1	Ref.
	Robust	6	122	0.28 (0.15–0.49)	<0.001 ***^,a^	0.40 (0.15–1.08)	0.068
Continuous Analysis							
	Age (per 5-year increase)	-	-	-	-	1.52 (1.14–2.03)	0.005 **
	BMI (per 1 kg/m^2^ increase)	-	-	-	-	0.78 (0.69–0.88)	<0.001 ***
	PA frequency (times/week)	-	-	-	-	0.94 (0.81–1.09)	0.421

OR = Odds Ratio; AOR = Adjusted Odds Ratio; CI = Confidence Interval; Ref. = Reference group; BMI = Body Mass Index; PA = Physical Activity. Statistical significance: * *p* < 0.05, ** *p* < 0.01, *** *p* < 0.001. * AOR was adjusted simultaneously for sex, age (continuous), education level, BMI (categorical), physical activity, nutritional status, and frailty status using the forced entry method. Multi-collinearity diagnostics demonstrated stable parameter estimates, with VIF metrics for all covariates strictly ranging between 1.08 and 1.45. To evaluate model robustness against potential residual confounding from unmeasured or under-measured clinical and socioeconomic parameters (such as detailed specific medication classes, cumulative comorbidity burden, and precise physical activity intensity metrics), alternative sensitivity analyses were integrated into the analytical framework. Predictors were tested across parallel continuous and categorical parameter models (e.g., modeling age and BMI concurrently as continuous scales versus discrete group factors). The high structural consistency and stability of the AOR across all iterations verified the resilience of the primary lifestyle, anthropometric, and nutritional determinants. ^a^ Calculated via Pearson’s Chi-square test. ^b^ Calculated via Fisher’s exact test due to a zero cell count (*n* = 0 in the non-sarcopenia cell for the ≥80 years age group). ^c^ The Crude Odds Ratio cannot be mathematically calculated due to division by zero; however, the strong baseline association was verified by Fisher’s exact test (*p* < 0.001), and model separation was accommodated in the multivariable framework (supported by continuous chronological age tracking).

## Data Availability

Data is contained within the article.

## Supplementary Materials

The following supporting information can be downloaded at https://www.mdpi.com/article/10.3390/ijerph23081040/s1. Table S1: Comparison of participant characteristics across the three study sites (*n* = 246).
