# Supplementary material for: Prevalence and Risk Factors of Sarcopenia Among Community-Dwelling Older Adults in Nakhon Si Thammarat, Thailand
Source: Int J Environ Res Public Health. 2026 Aug 10;23(8):1040. doi: 10.3390/ijerph23081040 (PMC13513653; doi:10.3390/ijerph23081040)
Supplement: Supplementary file 1 [file ijerph-23-01040-s001.zip › ijerph-4396920-supplementary.pdf]

Table S1. Comparison of participant characteristics across the three study sites ($n = 246$)

Characteristic / Variable	Total Cohort (n=246)	Tha Sala (*n*=85)	Pak Phanang (*n*=81)	Ron Phibun (*n*=80)	p-value †
**Socio-demographic Factors**					
**Age (years)**, *Mean* ± *SD*	69.4 ± 6.8	68.8 ± 6.5	70.1 ± 7.2	69.3 ± 6.7	0.412
Age group, *n (%)*					0.518
60 − 69 years	136 (55.3%)	50 (58.8%)	41 (50.6%)	45 (56.3%)	
70 − 79 years	92 (37.4%)	30 (35.3%)	34 (42.0%)	28 (35.0%)	
≥ 80 years	18 (7.3%)	5 (5.9%)	6 (7.4%)	7 (8.8%)	
**Sex**, *n (%)*					0.932
Female	218 (88.6%)	76 (89.4%)	71 (87.7%)	71 (88.8%)	
Male	28 (11.4%)	9 (10.6%)	10 (12.3%)	9 (11.3%)	
**Education Level**, *n (%)*					0.521
Primary school or uneducated	188 (76.4%)	62 (72.9%)	65 (80.2%)	61 (76.3%)	
Secondary school or higher	58 (23.6%)	23 (27.1%)	16 (19.8%)	19 (23.8%)	
**Marital Status**, *n (%)*					0.842
Married / Living with partner	152 (61.8%)	54 (63.5%)	48 (59.3%)	50 (62.5%)	
Single / Widowed / Divorced	94 (38.2%)	31 (36.5%)	33 (40.7%)	30 (37.5%)	
**Anthropometrics & Clinical Factors**					
**Body Mass Index (BMI, kg/m^2)**, *Mean* ± *SD*	22.8 ± 3.9	23.1 ± 3.8	22.4 ± 4.1	22.9 ± 3.8	0.485
BMI category, *n (%)*					0.624
Underweight (< 18.5 kg/m^2)	32 (13.0%)	9 (10.6%)	13 (16.0%)	10 (12.5%)	
Normal (18.5 − 22.9 kg/m^2)	112 (45.5%)	38 (44.7%)	36 (44.4%)	38 (47.5%)	
Overweight/Obese (≥ 23.0 kg/m^2)	102 (41.5%)	38 (44.7%)	32 (39.5%)	32 (40.0%)	
**Nutritional Status (MNA-SF)**, *n (%)*					0.612
Normal nutritional status	174 (70.7%)	62 (72.9%)	54 (66.7%)	58 (72.5%)	
At risk of malnutrition / Malnourished	72 (29.3%)	23 (27.1%)	27 (33.3%)	22 (27.5%)	
**Physical Activity Level**, *n (%)*					0.573
Regular physical activity (≥ 150 min/week)	130 (52.8%)	47 (55.3%)	39 (48.1%)	44 (55.0%)	
Inadequate / Inactive (< 150 min/week)	116 (47.2%)	38 (44.7%)	42 (51.9%)	36 (45.0%)	
**Sarcopenia Outcome**					
**Sarcopenia Prevalence (AWGS-2019)**, *n (%)*	68 (27.6%)	21 (24.7%)	25 (30.9%)	22 (27.5%)	0.671

† ***p*-values** were calculated using One-Way Analysis of Variance (ANOVA) or Kruskal-Wallis test for continuous variables, and Pearson's Chi-square test (χ^2) or Fisher's exact test for categorical variables across the three districts.

Abbreviations**:** *AWGS-2019* = Asian Working Group for Sarcopenia 2019 consensus criteria; *BMI* = Body Mass Index; *MNA-SF* = Mini Nutritional Assessment-Short Form; *SD* = Standard Deviation.
